# Variation in pelvic shape and size in Eastern European males: a computed tomography comparative study

**DOI:** 10.7717/peerj.6433

**Published:** 2019-02-20

**Authors:** Bartosz Musielak, Anna Maria Kubicka, Michał Rychlik, Jarosław Czubak, Adam Czwojdziński, Andrzej Grzegorzewski, Marek Jóźwiak

**Affiliations:** 1 Department of Pediatric Orthopedics and Traumatology, Poznań University of Medical Sciences, Poznań, Poland; 2 Department of Zoology, Poznań University of Life Sciences, Poznań, Poland; 3 Division of Virtual Engineering, Poznań University of Technology, Poznań, Poland; 4 Department of Orthopedics, Pediatric Orthopedics and Traumatology, Centre of Postgraduate Medical Education, Otwock, Poland; 5 Department of Orthopedics and Pediatric Orthopedics, Medical University of Lódź, Łódź, Poland

**Keywords:** Three-dimensional reconstruction, Geometric morphometrics, Medieval population

## Abstract

**Background:**

The significantly accelerated development of human society in the last millennium has brought about changes in human behavior and body mass that may have influenced human bone morphology. Our objective was to analyze the variation in pelvic shape and size in males from modern and medieval populations.

**Methods:**

We obtained 22 pelvic girdles of adult males from a medieval cemetery located in Cedynia, Poland. The control group comprised 31 contemporary male pelves from individuals inhabiting the same region. The analyzed parameters were: interspinous distance (ISD), intercristal distance (ICD), intertuberous distance (ITD), anatomic conjugate of the pelvis, height of the pelvis (HP), iliac opening angle (IOA), iliac tilt angle (ITA), and ISD/ITD/HP ratio. Geometric morphometrics was used to analyze differences in shape in the pelves. All analyses were carried out on three-dimensional CT reconstructions of pelves.

**Results:**

ISD, ICD, and IOA were significantly greater in modern pelves than in those from Cedynia, but no significant differences were seen between the two groups in ITD, anatomical conjugate, HP, or ITA. ISD/ITD/HP ratios were significantly lower in the Cedynia group. Geometric morphometrics revealed significant differences in pelvic shape between the analyzed groups.

**Discussion:**

The pelves of modern males are larger, wider, and flatter than those of medieval males. Changes in the set of daily activities that produce mechanical loading and estimated body mass may constitute the main factors explaining pelvic variability. However, differences in ontogenesis should also be taken into consideration, especially since growth in past populations is often found to be reduced relative to modern populations.

## Introduction

Many scientific articles analyzing variation in human pelvic shape have been published to date. However, there are several other factors that are crucial in shaping the morphology of the human pelvic girdle. The genetic (e.g., heritability) factor, activity, neutral processes (i.e., mutation, genetic drift, migration), number of offspring, and age are expected to explain variation in pelvic anatomy within and between populations (e.g., Schutz, Donovan & Hayes, 2009; Wells, DeSilva & Stock, 2012; Betti et al., 2013; Betti et al., 2014; Huseynov et al., 2016; Betti, 2017). Betti et al. (2014) showed that minimum temperature and precipitation also have an effect on the size and shape of the os coxae, indicating that individuals from cold environments exhibit a tendency towards larger pelvic bones. Another significant factor is nutritional deficiencies, which influence pelvic development and lead to a flatter pelvis (Wells, DeSilva & Stock, 2012).

The pelvic girdle is regarded as one of the most dimorphic elements in the human skeleton (e.g., Bruzek, 2002), though equally variable in both sexes (Tague, 1989). However, differences in pelvic shape and size between sexes appear after puberty, when female developmental trajectories start to diverge from the common course, resulting in increasing obstetric dimensions (Huseynov et al., 2016). Recent studies have also challenged the obstetric dilemma hypothesis, according to which the human pelvis reflects the demands of childbirth (e.g., Grabowski, 2013; Warrener et al., 2015). It has been hypothesized that increased obstetric dimensions allowing for relatively easy passage of larger-brained neonates could not be possible because this would increase energetic cost of bipedal walking and running (Grabowski, 2013). However, latest research showed that energy expenditure during locomotion is at the same level of efficiency in both sexes; therefore, the wider pelvic canal in females should not cause a significant rise in energy expended while walking or running (Dunsworth et al., 2012; Warrener et al., 2015).

Notwithstanding numerous research papers on coxal size and shape variation, little is known about the influence of human behaviors connected with the transition from agrarian to global economic production (e.g., from farming to urbanization) on this area in the human skeleton. We understand human behavior as the set of activities of daily living that produce mechanical loading and cause skeletal responses. The research has usually examined temporal trends in bone strength, assessing the cross-sectional geometry of the upper and lower limbs (e.g., Ruff et al., 2015; Kubicka et al., 2018). For example, Macintosh, Pinhasi & Stock (2017) showed an increase in the bone strength of the upper limbs in women associated with the beginning of agriculture and the intensification of sedentism. Other research has demonstrated a decrease in lower limb bone strength relative to body size, beginning in the Neolithic and continuing through the Iron/Roman period (Ruff et al., 2015). This may indicate that the pattern of change in skeletal parameters associated with the development of agriculture is still not clear (Auerbach, 2011). Ruff et al. (2015) suggested that change in bone strength is more closely associated with a systematic decline in mobility and less with increasing mechanization and urbanization; however, this finding relates to the upper and lower limbs, whereas little is known about changes in the size and shape of the axial bones such as the pelvis since the Industrial Revolution.

Along with the significantly accelerated technological development of the last millennium, especially with respect to automation, came many changes in diet, modes of transportation, daily activities, and occupations (Aleksandrova et al., 2014; Floud et al., 2011; Popkin, 2001; Zimmet, Alberti & Shaw, 2001). For example, mean walking distances have decreased with the advent of a more sedentary lifestyle (Agrawal & Schimek, 2007). Thus, the significant decline in the level of human physical activity and increasing mechanization (Popkin, 2005) may play a role in the development and variation of the human pelvis.

Greater body mass requires greater production of force by muscles (Kram & Taylor, 1990) and affects net joint movements associated with locomotion in humans (Hora et al., 2017). Reynolds & Hooton (1936) suggested that pelvic structure might respond to body posture and weight; however, as they wrote, their findings were suggestive only, due to methodological errors such as small sample size, incomplete data and problems with interpretations of drawings of the pelvis. On the other hand, the effect of body size on variability in human postcranial shape is still understudied and unclear. Nevertheless, it can be assumed that body mass can influence the remodeling of the bones of the lower limbs and pelvic girdle via the musculoskeletal complex.

Taking into account that the pelvic girdle is plastic during development (e.g., Huseynov et al., 2016; Kurki, 2017), the question thus arises as to whether changes in functional behavior and body weight have resulted in variations in pelvic shape, especially given that locomotion and body weight are transmitted to the lower limbs through the pelvis (DeSilva & Rosenberg, 2017) and that the human pelvis shows a high degree of variability within and between modern populations (Betti, 2017). Our attention was directed to the differences in the shape and size of the human pelvis between two populations, dating from different historical periods but ethnically uniform and deriving from the same geographical region (Poland). The first analyzed group contained intact pelvic bones from a medieval cemetery located in Cedynia, Poland, while the second included pelves of modern Polish males.

We decided to test the two following hypotheses: (1) men from a modern population are characterized by a pelvic shape different from that of individuals from the Middle Ages; (2) body mass influences the shape of the pelvic girdle in males. We analyzed only male pelves because we were thus able to focus on fewer factors potentially influencing bone shape than in the case of females, whose pelvic morphology changes during their lifetimes (e.g., Huseynov et al., 2016). The results of our study may help in the interpretation of functional variation of the pelvis due to different physical activities and body mass. Moreover, specific variations in the pelvic shape are associated with hip pain and its pathologies (Lewis et al., 2017); thus, understanding the influence of body mass on the pelvic shape may help in medical studies (e.g., Musielak et al., 2016). In order to achieve the main purposes presented above, we analyzed CT images of pelvic bones, using traditional measurements and geometric morphometrics. The combination of these two methods enabled a comprehensive demonstration of differences in pelvic shape.

## Materials and Methods

### Medieval population

The analyzed material consisted of 22 human male pelvic girdles obtained from a medieval cemetery located in Cedynia (Poland). The entire skeleton of each individual was assessed for any pathological changes such as fractures, osteophytes, porosity, or eburnation of articular surfaces. Only individuals with no observable pathological changes were accepted for further analysis. Sex was assessed based on the standards of Buikstra & Ubelaker (1994), which provides six aspects of skull morphology (nuchal crest, mastoid process, supraorbital margin, supraorbital ridge, glabella, mental eminence), and on the Phenice method (1969), based on the sexual dimorphism of pelvic morphology. Age was estimated using cranial suture closure (Buikstra & Ubelaker, 1994; White & Folkens, 2005a; White & Folkens, 2005b) and the auricular surface of the ilium (Lovejoy et al., 1985, mean age = 45.32 ± 9.46). To be sure that only pelvic girdles of individuals over 20 years old were used in the study, pelves with a completely fused iliac crest were chosen (Schaefer, Black & Scheuer, 2009). All samples were preserved in their entirety, without a pubic symphysis and with disconnected sacroiliac joints. Each pelvis had become separated into a sacral bone and two hemipelves.

The Cedynia necropolis served as the burial site for a group of 320 individuals within a span of some 9 generations (Torlińska-Walkowiak & Jerszyńska, 2011). The cemetery is dated to the period from the end of the 10th century till the first half of the 14th century (Myszka & Piontek, 2010; Piontek, Jerszyńska & Nowak, 2001). Based on archeological research, the economy of the medieval population from Cedynia was based on agriculture, animal husbandry, and crafts (Torlińska-Walkowiak & Jerszyńska, 2011; Iwaszczuk, 2014). The very low frequency of occurrence of Harris lines shows an absence of pathological and nutritional stress in this group (Piontek, Jerszyńska & Nowak, 2001). The prevalence of enamel hypoplasia in individuals from Cedynia fluctuates around 20% (Krenz-Niedbała, 2017), indicating the rare occurrence of physiological stress episodes during childhood. Compared to other European medieval populations, the analyzed group shows a low level of experience of stress events and nonspecific infections. Moreover, the medieval individuals constitute a homogenous group in terms of ethnicity and religion (Krenz-Niedbała, 2017).

### Modern population

The control group comprised 31 pelvic CT images of Polish males obtained between 2012 and 2014 at the Clinical Hospital in Poznań (Poland). The sex and age of the modern males were obtained from a hospital database. For this part, only pelves of individuals over 20 years old were used (mean age = 61.9  ± 15.87). Records were taken consecutively from patients diagnosed with nonorthopedic surgical conditions (e.g., suspected ileus) and with no pelvic bony lesions or inflammation. All patients in the control group were from Poland, the same region of Europe as the archeological cases. The research was part of a broader research project, based on CT examinations of human pelves, which was bioethically approved by the Poznań University of Medical Sciences Bioethical Committee (ethical approval no. 499/10). All of the participants in the study gave their verbal consent.

### Reconstruction of the pelvic girdle

Prior to diagnostic measurements, pelvic bones were reconstructed into a complete pelvic girdle of anatomic proportions using commercial glue and alignment of articular surfaces. The alignments were done manually, in such a manner that each pelvic girdle was connected by the whole area of the *facies auricularis* of the sacrum and pelves while maintaining the parallel position of surface joints. All samples underwent CT examination using a GE LightSpeed VCT 64-slice CT (GE Healthcare, Wauwatosa, WI, USA). Three-dimensional (3D) CT reconstructions were used to determine pelvic anthropometric parameters. The prepared model subsequently underwent CT examination using 0.63- or 1.25-mm slice thicknesses. The DICOM-formatted scans were transferred to ScanIP (Simpleware Ltd., Exeter, UK) computer-aided design (CAD) software for comprehensive processing of 3D-image data. Rhinoceros software (Robert McNeel & Associates; Seattle, WA, USA) was used to obtain measurements from spatial images.

### Pelvic dimensions

The sacral base (SB) plane was used here as a reference plane for further measurement (Fig. S1), because it is the only plane that focuses on the pelvic-spinal unit, which is extremely important to pelvic orientation in the upright position, an orientation subject to the evolutionary process (Boulay et al., 2014; Lazennec, Brusson & Rousseau, 2011; Legaye, 2009; Legaye et al., 2011; Le Huec et al., 2011; Lovejoy, 1988). The horizontal plane of reference is defined as a plane interpolated from a mesh of points (at least 150) located on the surface of the SB. The vertical plane is perpendicular to the horizontal and coincides with the geometric center of the SB (which is automatically set based on the previously applied mesh of points) and the midpoint of the line connecting the centers of the pubic tubercles (set based on 30 points marked on each pubic tubercle surface). Point 0 was set as the center of the SB, according to the technique of Jóźwiak et al. (2015).

**Figure 1:**
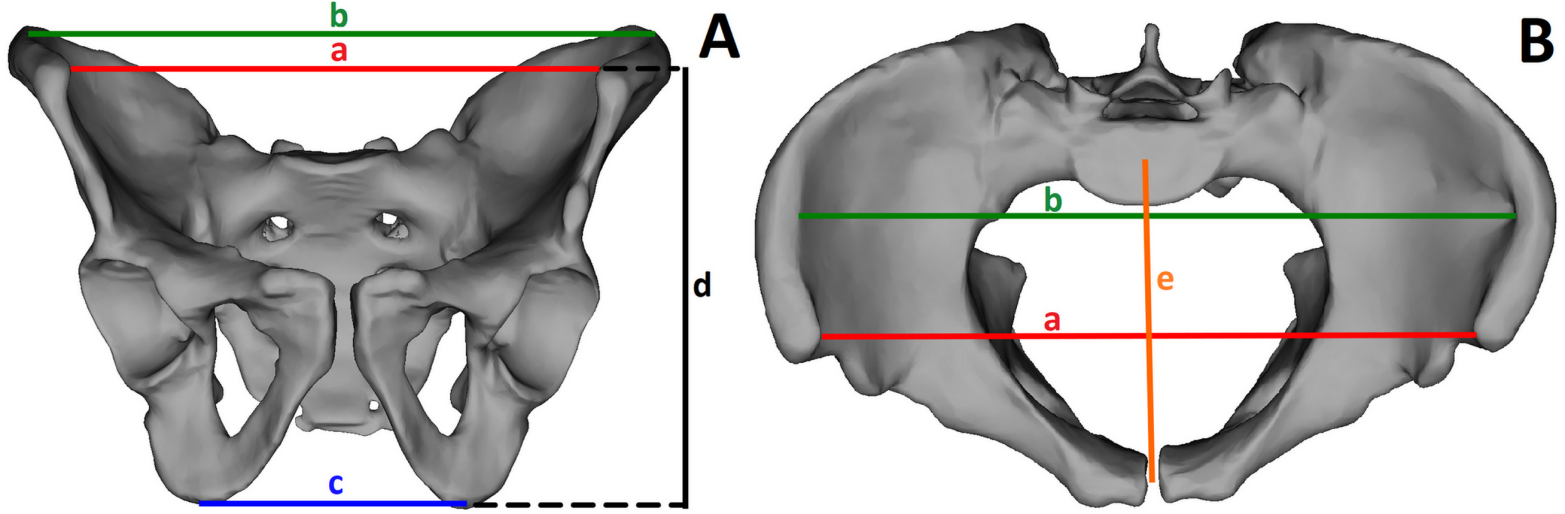
Analyzed parameters. (A) Anterior view of the male pelvic: a, interspinous distance (red line); b, intercristal distance (green line); c, intertuberous distance (blue line); d, height of the pelvis (black line); (B) Superior view of the male pelvic: a, interspinous distance (red line); b, intercristal distance (green line); e, anatomical conjugate (orange line).

**Figure 2:**
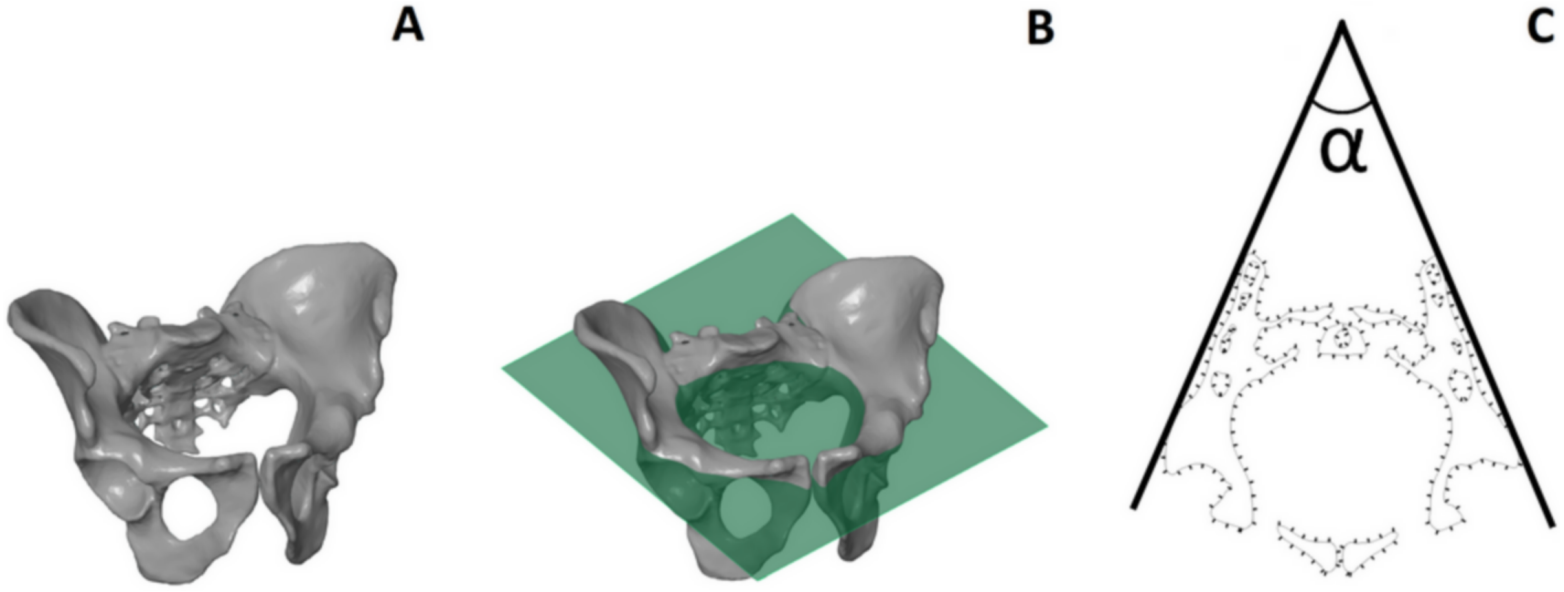
Iliac Opening Angle (IOA), measured in the inlet plane. (A) View of the male pelvic; (B) the male pelvic with the plane parallel to the inlet plane; (C) the angle formed by the lines tangential to the posterolateral wall of the iliac bones, measured in the inlet plane.

**Figure 3:**
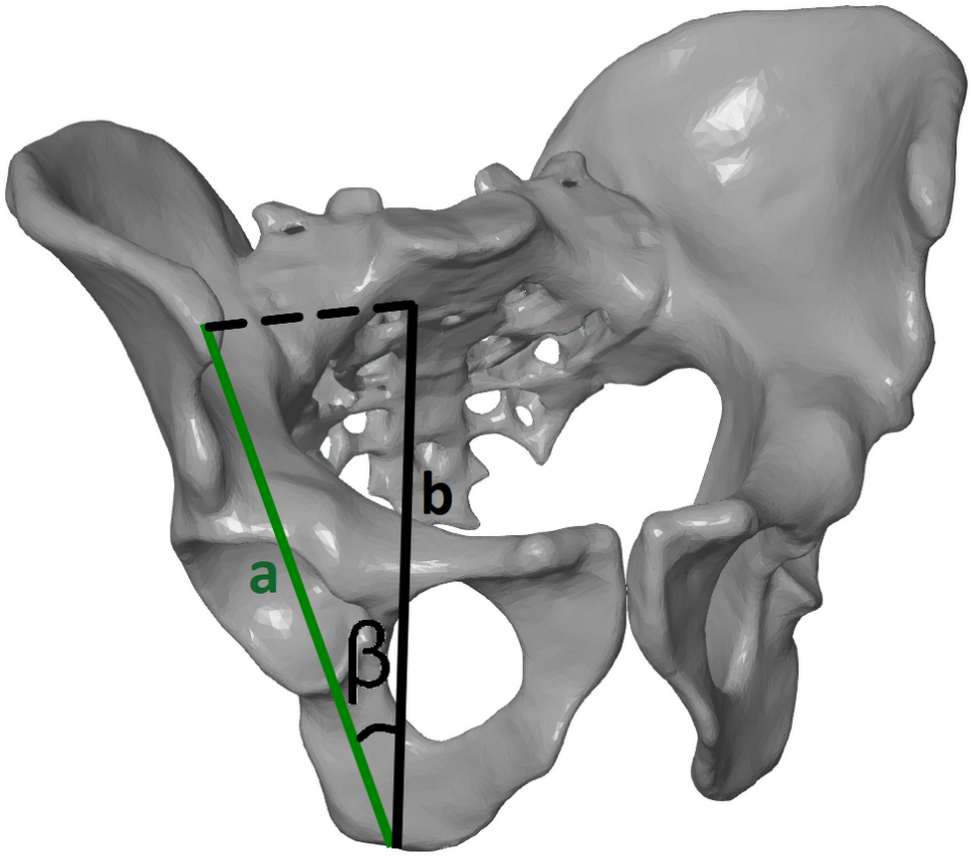
Tilt angle (ITA), measured in the frontal plane of the pelvis. The angle formed by the line connecting the anterior superior iliac spine point with the ischial tuberosity center (A) and the line between the projection of the anterior superior iliac spine on the sagittal plane (used for HP) and the ischial tuberosity center (B).

The following parameters were measured based on the pelvic spatial models: interspinous distance (ISD), intercristal distance (ICD), intertuberous distance (ITD), anatomic conjugate of the pelvis, height of the pelvis (HP), iliac opening angle (IOA), iliac tilt angle (ITA), and ISD/ITD/HP ratio (Fig. 1, Table 1). To assess the shape of the pelvis, we used the ratio of ISD to ITD to HP, which describes the span of the iliac wings (Figs. 2 and 3).

**Table 1:** Description of the pelvic dimensions.

**Measurements**	**Description**
**Interspinous Distance (ISD)**	The distance between the two most prominent points of the anterior superior iliac spine (ASIS) in the sagittal plane
**Intercristal distance (ICD)**	The longest distance between the most prominent points of the iliac crest; the line between these points is parallel to the frontal plane of the pelvis
**Intertuberous Distance (ITD)**	The distance between the lowest points of the ischial tuberosities in the sagittal plane
**Anatomic Conjugate of the Pelvis**	The distance between point 0 of the pelvis and the midpoint of the line connecting the centers of the pubic tubercles
**Height of the Pelvis (HP)**	The relative distance between the ASIS point (projected onto the sagittal plane passing through the ischial tuberosity point) and the ischial tuberosity point (also projected)
**Iliac Tilt Angle (ITA)**	The angle formed by the line connecting the ASIS point with the ischial tuberosity center (ITC) and the line between the projection of ASIS on the sagittal plane (used for HP) and the ITC
**Iliac Opening Angle (IOA)**	The angle formed by the lines tangential to the posterolateral wall of the iliac bones, measured in the inlet plane

The parameters HP, ITA, and ISD/ITD/HP ratio were measured separately for the left and the right side of each individual, whereas all other parameters were taken for the pelvis as a whole. Some of the parameters measured in the present study (ISD, ICD, ITD, HP) are commonly used in clinical practice and medical research; however, we were unable to find their exact descriptions used with 3D images (Boyle et al., 2005; Decking et al., 2006; Freed et al., 2000; Hoyte et al., 2005; Stewart, Cowan & Philpott, 1979). We therefore considered it appropriate to define and precisely measure these distances to obtain high-quality results. All of the above-mentioned parameters were used recently by Musielak et al. (2016) to evaluate the intrinsic relationship between the structures and positions of the pelvis and acetabulum.

### Pelvic shape

To analyze differences in pelvic shape between medieval and modern males, we used geometric morphometric methods. One 3D reconstruction of a pelvic girdle with average dimensions was set as a template on which 46 landmarks and 110 sliding semilandmarks were manually distributed (Table S1, Fig. S2). In order to minimize differences in semilandmark distribution, a thin plate spline (TPS) function was applied, enabling the interpolation of semilandmarks between the template and each sample based on manually distributed landmarks. Thus, all semilandmarks were automatically warped from the template to each individual girdle, based on landmarks using the TPS function (see details in O’Higgins et al., 2012).

The digitized landmarks and semilandmarks were chosen according to Huseynov et al. (2016); however, we decided to use fewer homologous points due to our limited sample size. The location of landmarks and semilandmarks on the pelvic girdle, along with their descriptions, can be found in the Fig. S2 and Table S1. The process of digitization was carried out by a single observer experienced in geometric morphometric analysis using EVAN Toolbox software (version 1.71 for Windows).

### Estimation of body mass

Mechanical methods were used to assess the body mass of each analyzed individual. The anterior-posterior breadth of the femoral head was measured on each side of each individual and then averaged. This enabled us to remove bias caused by lateralization. We used the dimensions of the femoral head to estimate body mass because its articular surface is little subject to the influence of differences in activity level and muscular loadings (Auerbach & Ruff, 2004). Femoral head measurement in the medieval group was taken by a single individual using a digital dial caliper. The femoral head parameter in the contemporary samples was measured by the same individual, based on 3D femoral reconstruction. Three body mass estimates based on previously published equations (Ruff, Scott & Liu, 1991; McHenry, 1992; Grine et al., 1995) were obtained for each individual and averaged (Auerbach & Ruff, 2004).

### Statistical analysis

The Fleiss *κ* coefficient and Intraclass Correlation Coefficients (ICCs) were used to assess inter- and intra-rater reliability, respectively. Intra-rater reliability was calculated for one observer. The geometric parameters measured on the spatial models were assessed for reliability. Thirty 3D pelvic reconstructions randomly chosen from both groups (15 from each group) were assessed twice by two investigators (one advanced, one novice) at intervals of at least 6 months.

A variety of methods are currently available to quantify and analyze variation in bones (e.g., Chintalapani et al., 2007; Kubicka et al., 2016; Betti, 2017); however, we based our analyses on linear and geometric morphometrics. Pelvic measurements in the Cedynia and control groups were tested for normality and homogeneity of variance using the Shapiro–Wilk test and Levene’s test, respectively. All variables were normally distributed and characterised by homogeneity of variances; thus, Student’s *t*-test was used to compare the average values of pelvic measurements of the Cedynia and control groups. Statistical analyses were performed with STATISTICA 12 software (StatSoft, Inc., Tulsa, OK, USA).

Generalized Procrustes analysis was carried out in order to superimpose the pelvic girdle. This procedure, applied in order to transform the data into linearized Procrustes shape space, consisted of three stages: translation (averaging the position of landmarks), scaling to one size, and rotation (minimizing the summed squared distances between landmarks; e.g., Zelditch, Swiderski & Sheets, 2012). Next, a multivariate regression test was performed to determine whether pelvic shape correlates with size. This type of association is called allometry and can be a crucial component of shape variation (Outomuro & Johansson, 2017). It is tested by checking the association between the Procrustes coordinates and centroid size, a measure of geometric scale calculated as the square root of the summed squared distances of each landmark from the center of the landmark configuration (Zelditch, Swiderski & Sheets, 2012). Next, Principal Component Analysis (PCA) was carried out in order to simplify the dimensionality of the shape space and to show the major patterns of pelvic shape variation in medieval and modern males. Additionally, we used a Procrustes ANOVA test to analyze the significance of differences between the pelvic girdles of the medieval and modern males. Multivariate regression was performed to analyze pelvic shape associated with estimated body mass. Geometric morphometric analyses were carried out using MorphoJ (version 1.06d, Klingenberg, 2011) and EVAN Toolbox (version 1.71). The significance level for all statistical analyses was *p* < 0.05.

## Results

All results concerning inter- and intra-rater reliability of the parameters describing the pelvic dimensions are presented in Table 2. The Fleiss *κ* coefficients indicated good and excellent inter-observer reproducibility, while the ICC values demonstrated mostly excellent intra-rater reliability in the determination of all of the pelvic parameters.

**Table 2:** Intra- and inter-rater reliability assessment of anthropological parameters used in the study.

	Intra-rater reliability		Inter-rater reliability	
	Intraclass correlation		Fleiss *κ*	
	Coefficient		Coefficient	
Interspinous Distance (ISD)	**0.73**	Good	**0.80**	Excellent
Intercristal distance (ICD)	**0.92**	Excellent	**0.85**	Excellent
Intertuberous Distance (ITD)	**0.83**	Excellent	**0.88**	Excellent
Anatomic Conjugate of the Pelvis	**0.95**	Excellent	**0.89**	Excellent
Height of the Pelvis (HP)	**0.67**	Good	**0.82**	Excellent
Iliac Tilt Angle (ITA)	**0.92**	Excellent	**0.77**	Excellent
Iliac Opening Angle (IOA)	**0.80**	Excellent	**0.70**	Good

**Notes.**

Bold indicates significant coefficients at level *p* < 0.05.

The characterization of coefficient reliability based on a quartile system: poor (0–0.24), moderate (0.25–0.49), good (0.50–0.74), excellent (0.75–1.00).

All measurements are presented in Table 3. Mean ISD, ICD, and IOA were significantly greater in the control group than in the Cedynia group (*p* = 0.001, 0.048, and 0.002, respectively). However, no differences were observed between the two groups in ITD (*p* = 0.411), anatomical conjugate (*p* = 0.176), HP (*p* = 0.096), or ITA (*p* = 0.152). The ISD/ITD/HP ratio was significantly lower in the Cedynia group (*p* = 0.007).

**Table 3:** Comparison of anthropometric measurements of pelves between modern (*n* = 31) and medieval (*n* = 22) group.

	Group	Mean value	SD	Minimum value	Maximum value	*t*	*p* value
*Distance* [mm]							
ISD	Modern	**251.56**	18.32	210.77	295.94	−3.711	**0.001**
	Medieval	**234.31**	15.13	209.48	262.56		
ICD	Modern	**275.06**	18.25	226.14	307.54	−2.019	**0.048**
	Medieval	**264.41**	19.83	220.12	298.52		
ITD	Modern	105.91	11.50	82.14	124.50	−0.738	0.411
	Medieval	103.39	9.99	85.83	124.13		
Anatomic conjugate	Modern	125.71	14.41	98.40	153.90	1.371	0.176
	Medieval	130.59	9.92	113.55	146.75		
HP	Modern	151.92	16.81	99.17	184.95	1.179	0.096
	Medieval	156.33	10.14	137.26	175.02		
*Angle (degrees)*							
IOA	Modern	**25.47**	4.97	14.59	35.40	−2.981	**0.002**
	Medieval	**22.53**	4.29	12.87	32.17		
ITA	Modern	52.86	9.56	27.86	69.71	1.038	0.152
	Medieval	49.20	8.21	30.85	63.12		
*Ratio*							
ISD/ITD/HP	Modern	**0.0161**	0.0033	0.0114	0.0246	−2.529	**0.007**
	Medieval	**0.0147**	0.0020	0.0110	0.0180		

**Notes.**

Statistically significant differences are marked in bold.

ISD interspinous distance ICD intercristal distance ITD intertuberous distance HP height of the pelvis IOA iliac opening angle ITA iliac tilt angle SD standard deviation *t* value for Student’s *t*-test

**Figure 4:**
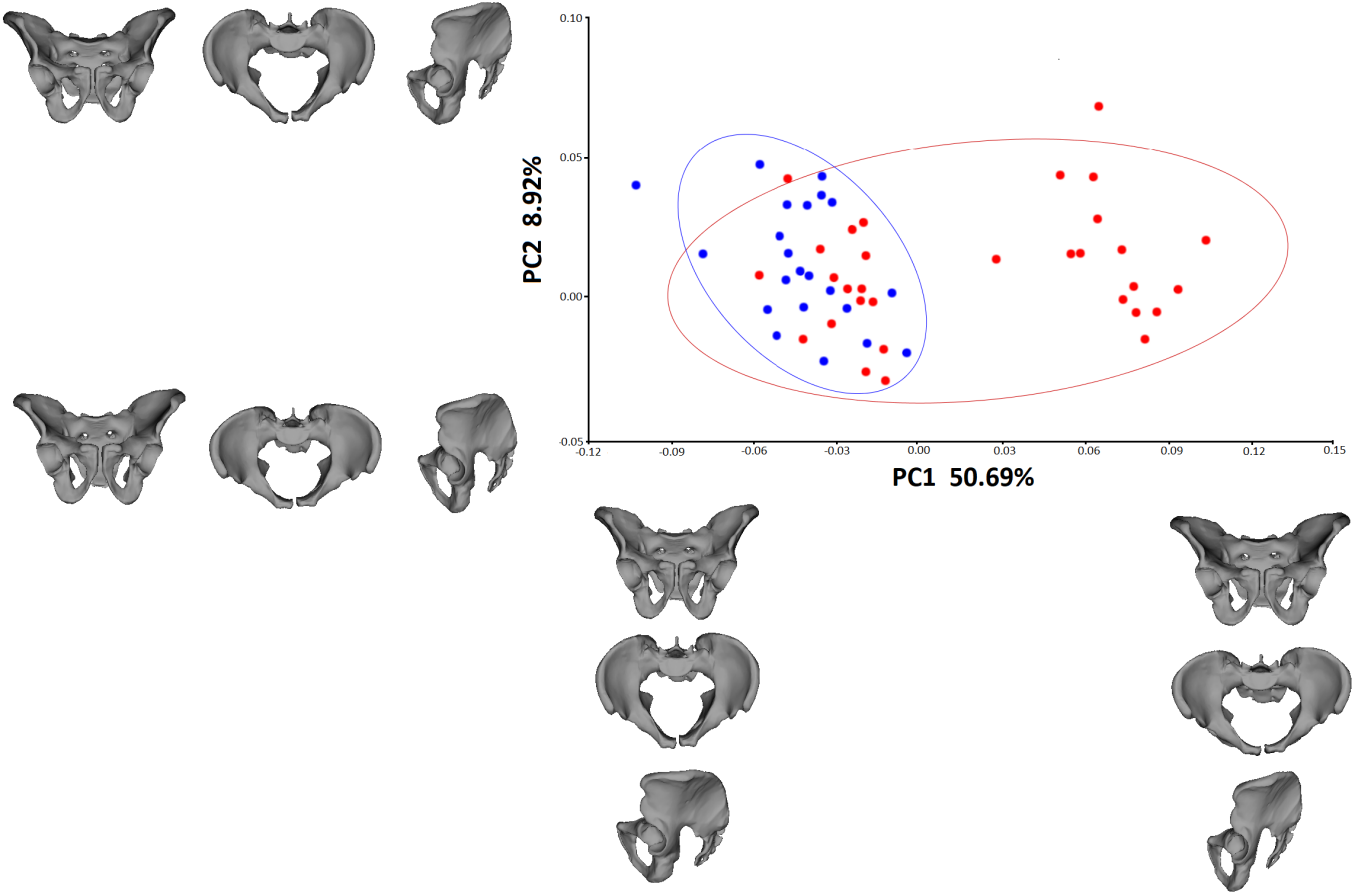
PCA of the pelvic shape in males from the medieval and modern groups. Blue dots—medieval males, red dots—modern males.

**Figure 5:**
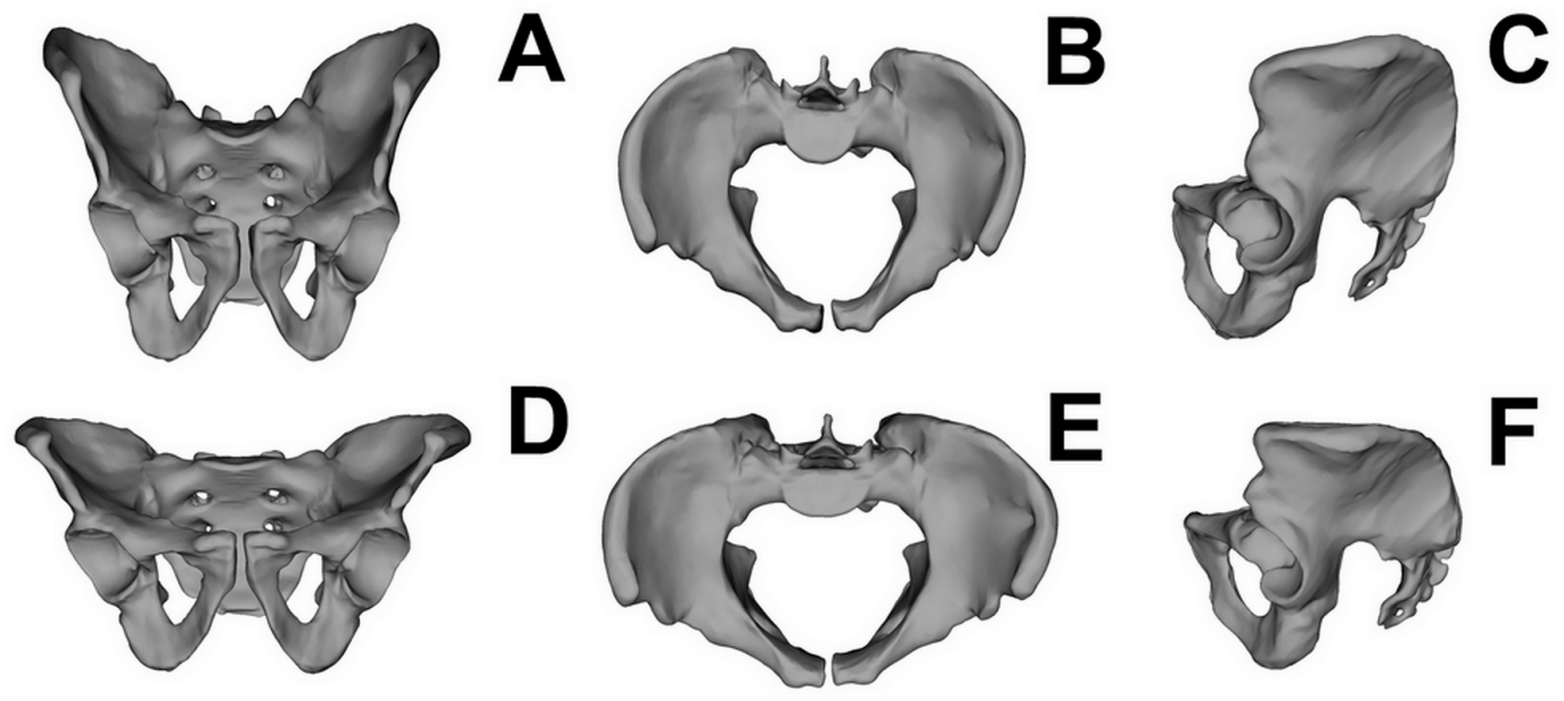
Mean shape of the pelvic girdle. (A) Anterior view of the male pelvic from the medieval population; (B) superior view of the male pelvic from the medieval population; (C) lateral view of the male pelvic from the medieval population; (D) anterior view of the male pelvic from the modern population; (E) superior view of the male pelvic from the modern population; (F) lateral view of the male pelvic from the modern population.

**Figure 6:**
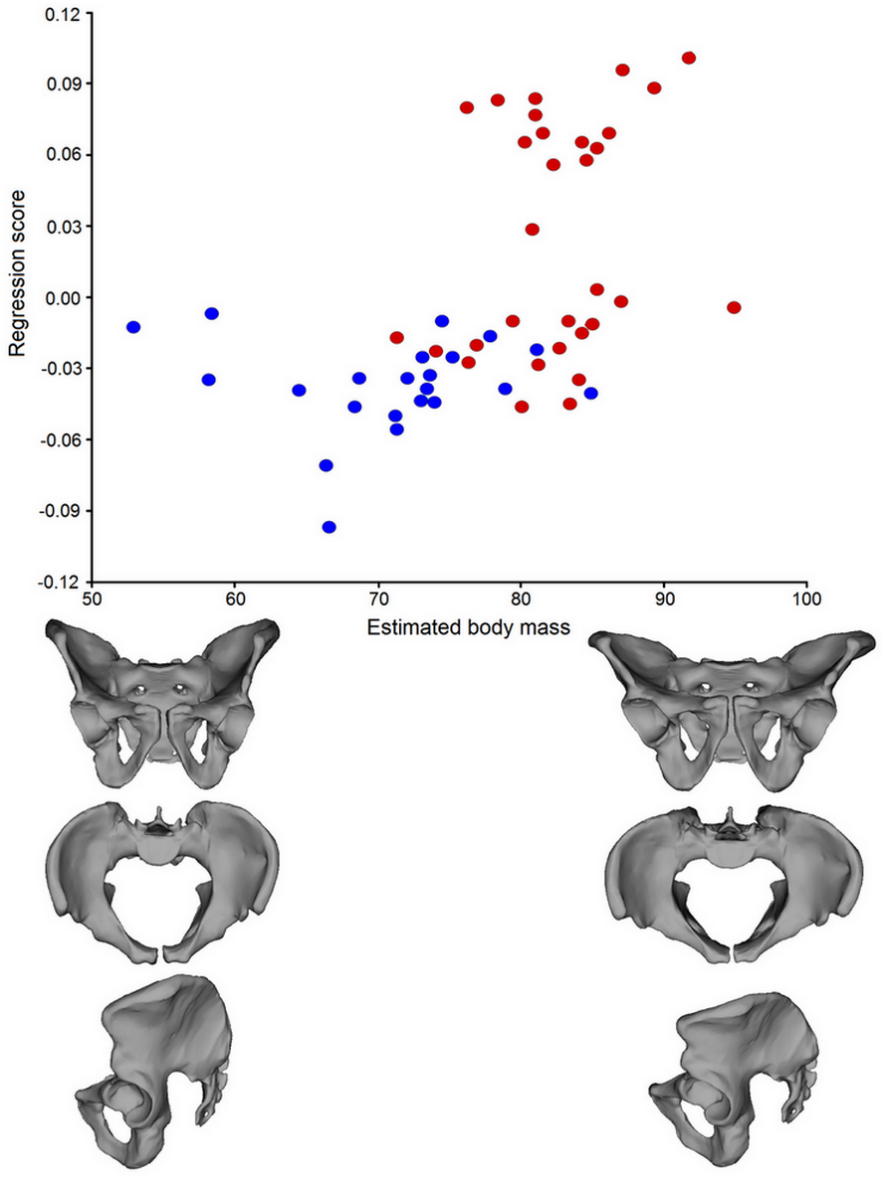
A scatterplot of pelvic shape and estimated body mass (based on the diameter of the femoral head) in males, derived from a multivariate regression between these two sets of variables. Blue dots—medieval males, red dots—modern males.

Regression testing revealed that the association between pelvic girdle shape and centroid size was not significant (*p* = 0.0654, predicted percentage = 4.25%); therefore, further geometric morphometric analysis was carried out on Procrustes coordinates, but without accounting for the allometric component. The PCA showed that the first two PCs were responsible for major variations in pelvic shape (Fig. 4). PC1 and PC2 explained 50.69% and 8.92% of variation, respectively. Individuals with positive PC1 values were characterized by a less upright ilium than those with negative values. Males with positive PC2 values were characterized by a more perpendicular acetabulum (in relation to the transverse axis). The medieval males exhibited markedly less variance than the individuals from the modern population in terms of PC1. The Procrustes ANOVA test showed significant differences in pelvic shape between the medieval and modern groups (*p* = 0.0001), but no significant differences in centroid size between these groups (*p* = 0.8744). Figure 5 shows that the pelvic girdle of the medieval male was characterized by a more upright ilium, a slightly narrower pelvic inlet, a less obtuse subpubic angle, and a more perpendicular acetabulum than those of the modern male. Moreover, regression testing revealed that the shape of the pelvic girdle and diameter of the femoral head (i.e., estimated body mass) were significantly interrelated (*p* = 0.0006, predicted percentage = 14.07%). Figure 6 shows that an individual with greater diameter of the femoral head (i.e., estimated body mass) is characterized by a pelvis with a more perpendicular ilium, an oval pelvic inlet, and an acetabulum directed downwards.

## Discussion

Our results support the first hypothesis that pelvic shape and size differ significantly between the two groups. The modern males are characterized by a significantly greater distance between the two most prominent points of the anterior superior iliac spine (ISD), a greater distance between the most prominent points of the iliac crest (ICD), and a larger angle measured in the inlet plane (IOA) than medieval individuals. The modern group is also characterized by greater values of the ISD/ITD/HP ratio, which means that the ilia of contemporary males are less upright and that their pelves are lower than those of the medieval farmers. The observed changes in the pelvic girdle measurements are generally consistent with the geometric morphometric analysis. The pelves of the medieval males are characterized by a more upright ilium, narrower pelvic inlet, and more perpendicular acetabulum than those of the contemporary males.

Since many factors play a role in the final form of the pelvic girdle, it is difficult to determine which has the greatest influence. The observed pelvic morphological variation may reflect the functional demands on both groups and may be associated with the level and type of functional human behavior (a set of daily physical activities). Remodeling of bone shape results from mechanical loading (Ruff, Holt & Trinkaus, 2006; Verbruggen & Nowlan, 2017); thus, the increasing modularity of pelvic parts reflects changes in functional demands during an individual’s lifetime (Huseynov, Ponce de León & Zollikofer, 2017). Judging from archeological findings, medieval males in Poland were commonly involved in carrying loads, fishing and agriculture, and building operations such as chopping (Górecki, Wrzesiński & Łastowiecki, 1994; Torlińska-Walkowiak & Jerszyńska, 2011; Iwaszczuk, 2014). These kinds of physical activity involve mainly the upper limbs, but also produce high levels of mechanical loading (Kubicka et al., 2016) which is transferred to the lower limbs through the pelvis. In comparison, a constant reduction in physical activity among adults is currently being observed in Poland (Bergier et al., 2012). Accordingly, these behavioral differences may influence variation in pelvic shape and size, especially given that the influence of activity levels on pelvic form has been shown by other studies (e.g., Forwood & Burr, 1993; Abitbol, 1996; Hughes et al., 2002).

In addition to behavioral differences, the level of sedentary lifestyle can be another factor explaining pelvic morphology. Ruff et al. (2015) showed that changes in the bending strength of the femur and tibia are associated with a decline in mobility. Moreover, pelvic breadth and stature influence the cross-sectional properties of the femur (Ruff et al., 2015). Thus, a more sedentary lifestyle can partially explain the lowering of the ilium and widening of the pelvic inlet in the modern males.

Mechanical loading initiates skeletal remodeling (Ruff, Holt & Trinkaus, 2006; Verbruggen & Nowlan, 2017), although the ontogenetic development of the human pelvis can also be an important factor in pelvic shape variability. Skeletal development in past human populations is often found to be slower and reduced than that in modern societies (e.g., Wall, 1991; Mays, 1999; Mays, 2002; Šereikiene & Jankauskas, 2004). For example, Krenz-Niedbała (2017) showed that growth in Polish medieval and postmedieval children (including those in Cedynia) was reduced relative to modern standards due to unstable living conditions and environmental and sociocultural constraints in the Middle Ages. Many episodes of environmental stress at the age of 9–11 years could also contribute to a growth retardation of the population from Cedynia (Piontek, Jerszyńska & Nowak, 2001). Since the presented study focuses on the pelvic size, shape, and estimated body mass of adult individuals, implied differences in growth between the medieval and modern populations should be also taken into consideration.

Another possible explanation for the changes in pelvic shape is the tendency of the modern male to be characterized by greater estimated body mass than the medieval male, as shown by multivariate regression (see Fig. 6). The geometric morphometric analysis confirmed the second hypothesis, showing a significant association between estimated body mass and the shape of the pelvic girdle. Males with greater estimated body mass are characterized by a pelvis with a more horizontal ilium, an oval pelvic inlet, and an acetabulum directed downward. It is assumed that the most important mechanical function of the pelvic girdle is to transmit gravitational forces deriving from the weight of the head, trunk, and upper limbs to the lower extremities and ground (Tardieu et al., 2013). Moreover, the human pelvic girdle transfers loads from the trunk to the lower limbs (Verbruggen & Nowlan, 2017). Therefore, the need to provide support for heavier abdominal organs may be responsible for the observed changes in the pelves of modern males.

According to the PCA, the distribution of the modern data is unusual. The modern males, though showing much greater variance in pelvic shape than the medieval males, are divided into two subgroups along PC1. One portion of modern males is characterized by PC1 values similar to those of the medieval group; these values indicate an upright ilium. The other portion of modern samples exhibits positive PC1 values (Fig. 4). This division cannot be a consequence of incorrectly assessed sex, because the age and sex of each member of the modern group was known from the hospital database. The data distribution in question may be partly a consequence of disparities in the sample sizes of these two groups, as the modern population was more numerous than the medieval sample. On the other hand, the wide variation in the modern males can be partially explained by observed changes in pelvic morphology at the individual and population levels (e.g., Tague, 1989; Huseynov et al., 2016; Delprete, 2017). Nevertheless, the data distribution of the modern group suggests the need for further research on other contemporary populations.

Pelvic shape differentiation in males cannot be explained as a consequence of differences in body stature, as we found that base of the sacrum area and overall pelvic size computed as centroid size were similar in the medieval and contemporary samples. Body stature was similar in these two groups: the average body height was 175 cm for males from Cedynia (Myszka, 2017), 178.3 cm for modern Polish men (Kołodziej et al., 2015). Moreover, the observed differences are not a result of pelvic allometry (the correlation between pelvic shape and size), as demonstrated by non-significant regression analysis (*p* > 0.05). A slightly different result was obtained by Fischer & Mitteroecker (2017), who showed an allometric component in pelvic dimensions unrelated to birth, such as height-to-width ratio and orientation of the iliac blades. This discrepancy may be due to the comparison of both sexes in the cited study, whereas we analyzed only males.

According to Betti et al. (2014), variation in the pelvic girdle shows a neutral demographic history signature similar to that of the cranium. This may suggest that changes in the gene pool over the past millennium might be partially response for the observed differences in pelvic shape and size. However, the analyzed groups came from the same geographical region, one that from the Roman Iron Age until the present has exhibited the genetic continuity of certain matrilineages (Juras et al., 2014). Moreover, no radical climate changes have occurred in the European continent over the past thousand years. Thus, climate and genetic diversity can be excluded as main factors explaining the observed changes in pelvic shape and size. The diet of the two human groups probably had a slight influence on this variation, as Wells, DeSilva & Stock (2012) showed that nutritional deficiencies lead to developmental disturbance and flatter pelves, whereas medieval males were characterized by an upright ilium and narrow inlet.

This study is characterized by certain limitations. First, it involves a comparison of two groups of pelvic specimens categorized by time period but lacking an intermediate group of pelves that might have better demonstrated an association between estimated body mass and pelvic shape. Second, the Cedynia pelves were reconstructed from unearthed bones. Pelves are among the most robust elements of the postcranium (Verbruggen & Nowlan, 2017) and in this case were assembled using the highest standards; nevertheless, differences in their preservation may have had an influence on our results. Another limitation is associated with sex assessment of the medieval group. This part of the research was based on morphological traits of the skull and pelvis using standardized methods (Buikstra & Ubelaker, 1994; White & Folkens, 2005a; White & Folkens, 2005b). No method provides 100% accuracy in assessment; thus, there is a small likelihood of incorrect sex classification. On the other hand, the PCA showed that the medieval males are a rather homogeneous group in the context of pelvic shape, characterized by pelves with marked male traits.

Another limitation is the small sample size of the analyzed groups. The medieval males were selected from a large osteological collection; however, the very good state of preservation of the pelvic girdle (pelves and sacrum) required for this study limited the sample size of the group. Only patients diagnosed with nonorthopedic surgical conditions and with no pathological changes in the pelvic girdle could be included in the study. Even given the extensive hospital database, not many modern males fulfill these criteria. The differences in the level of preservation of the bones of the medieval group may have biased the group towards individuals with more robust pelvic morphology. Moreover, the age distribution of the groups were not the same, the modern population contained a greater number of older individuals than the medieval. On the other hand, the influence of this factor on pelvic shape may be slight, since we classified only individuals without degenerative changes. Taking these limitations and the unusual data distribution of the modern males into account, the conclusions should be viewed with caution, and similar studies should be carried out on other human populations.

Moreover, the study does not focus on sex differences, since only male pelves were analyzed. Pelvic morphology changes during the lifetime of a female (Huseynov et al., 2016) along with the number of offspring (Kurki, 2007); thus, it is important to carry out analyses using a large sample size. This study made use of an abundant medieval collection which, nevertheless, included very few well-preserved female pelves. On the other hand, the exclusive focus on males meant that we had to account for fewer factors that might have influenced the observed changes in bone shape.

In our opinion, analysis of pelvic dimensions using both traditional methods of measurement and geometric morphometrics may have had some advantages. For example, the combination of these two methods enabled Ward, Maddux & Middleton (2018) to carry out a sophisticated analysis of variation in pelvic anatomy related to overall torso forms among anthropoid taxa. Geometric morphometric analysis is an important tool in analyzing shape variation and covariation of shape with other variables and is now more accessible than ever (e.g., Cooke & Terhune, 2015). On the other hand, it is also time-consuming, and requires visualization of the objects. Thus traditional measurements taken with an electronic caliper may be a good solution when working with large osteological collections, or in field laboratories. Accordingly, we decided to apply two approaches (traditional measurements and geometric morphometrics) in order to determine whether they are mutually consistent. Our study shows that, while some conventional linear measurements did not differ significantly between the medieval and modern males, geometric morphometric analysis exhibited clear differences in pelvic shape between these two groups. This shows that conventional measurements (distance measures, angles, or ratios) used in this study underestimated the intra- and intergroup differences, probably due to a lack of ability to characterize the entire shape of an object. Another reason may be the analysis of parameters independently rather than as a part of a structure that may be subject to covariation (Cooke & Terhune, 2015). Therefore we suggest that geometric morphometrics be used in biological research in support of traditional measurements rather than independently.

Our measurements were performed using an original method of evaluation based on 3D CT reconstruction, a precise measurement tool whose reliability has been confirmed by previous studies (Jóźwiak et al., 2015; Musielak et al., 2016) as well as by high ICC values for intra- and inter-rater reliability. Geometric morphometric analysis was also carried out on 3D CT pelvis reconstructions using the Thin Plate Spline function, which enabled the interpolation of semilandmarks between the template and each sample. This approach also ensured a low level of error associated with landmark and semilandmark digitization (Kubicka et al., 2016).

We believe that the presented findings enable a superior and more accurate understanding of pelvic morphological variation. The results may be important in forensic anthropology, especially considering that the pelvic shape of the contemporary Polish male includes some female traits such as a horizontal ilium, obtuse subpubic angle, and an anteriorly-facing acetabulum (Buikstra & Ubelaker, 1994; White & Folkens, 2005a; White & Folkens, 2005b). This finding is important, as some research shows that the sexually dimorphic traits of the pelvis are quite stable across human populations (e.g., Velemínska et al., 2013) and that the pelvis is the best anatomical structure to use in determining an individual’s sex from its skeleton (e.g., White, Black & Folkens, 2012; Velemínska et al., 2013). The demonstrated association between estimated body mass and pelvic shape calls this into question to some extent, especially since it has been shown that some parts of the pelvic girdle, such as pelvic inlet shape, are less dimorphic than previously thought (Delprete, 2017). Finally, the obtained results may be also helpful in future orthopedic and pediatric studies, since evolutionary medicine has proved that knowledge of differences between past and contemporary environments is necessary for the solution of medical issues (e.g., Trevathan & McKenna, 1994).

After the age of 40, the female pelvis is significantly reduced in terms of obstetric dimensions due to the pubertal rise and premenopausal fall of estradiol levels (Huseynov et al., 2016). With age, the ilium in females becomes less upright, which explains certain changes in female pelvic girdle shape observed by Huseynov et al. (2016). However, these changes may also be associated with body mass and shifts in physical activity, as observed in a previous study (Hughes et al., 2002). Taking this into account, an additional study analyzing the association between habitual behavior, body mass, and female pelves would be useful.

## Conclusions

Linear measurements and geometric morphometrics show significant changes in pelvic shape and size between medieval and modern males. In our opinion, the main factors responsible for the differences in pelvic shape and size are changes in the set of activities of daily living and estimated body mass between medieval and modern males. Moreover, growth in past populations is often found to be reduced relative to modern populations; thus, differences in ontogenesis should also be considered as a factor. This variation cannot be explained as a consequence of differences in body stature, since both populations were characterized by similar body heights. Moreover, geographic and genetic variables can also be excluded, since both groups came from the same region and no radical climate changes have occurred in the European continent over the past thousand years. In future studies, similar research is needed to analyze changes in female pelves.

##  Supplemental Information

10.7717/peerj.6433/supp-1 Figure S1 Set of planes generated from the Sacrum Base using the XYZ coordinate system

10.7717/peerj.6433/supp-2 Figure S2 A pelvic girdle with landmarks (red dots) and semilandmarks (blue dots) (A) anterior view of the male pelvis; (B) superior view of the male pelvis; (C) lateral view of the male pelvis.

10.7717/peerj.6433/supp-3 Table S1 Definition of landmarks LM –landmark number

## Additional Information and Declarations

### Data Availability

The following information was supplied regarding data availability: MorphoSource: https://www.morphosource.org/Detail/ProjectDetail/Show/project_id/589.
