# Supplementary figures and images for: Variation in pelvic shape and size in Eastern European males: a computed tomography comparative study
Source: PeerJ. 2019 Feb 20;7:e6433. doi: 10.7717/peerj.6433 (PMC6387581; doi:10.7717/peerj.6433)

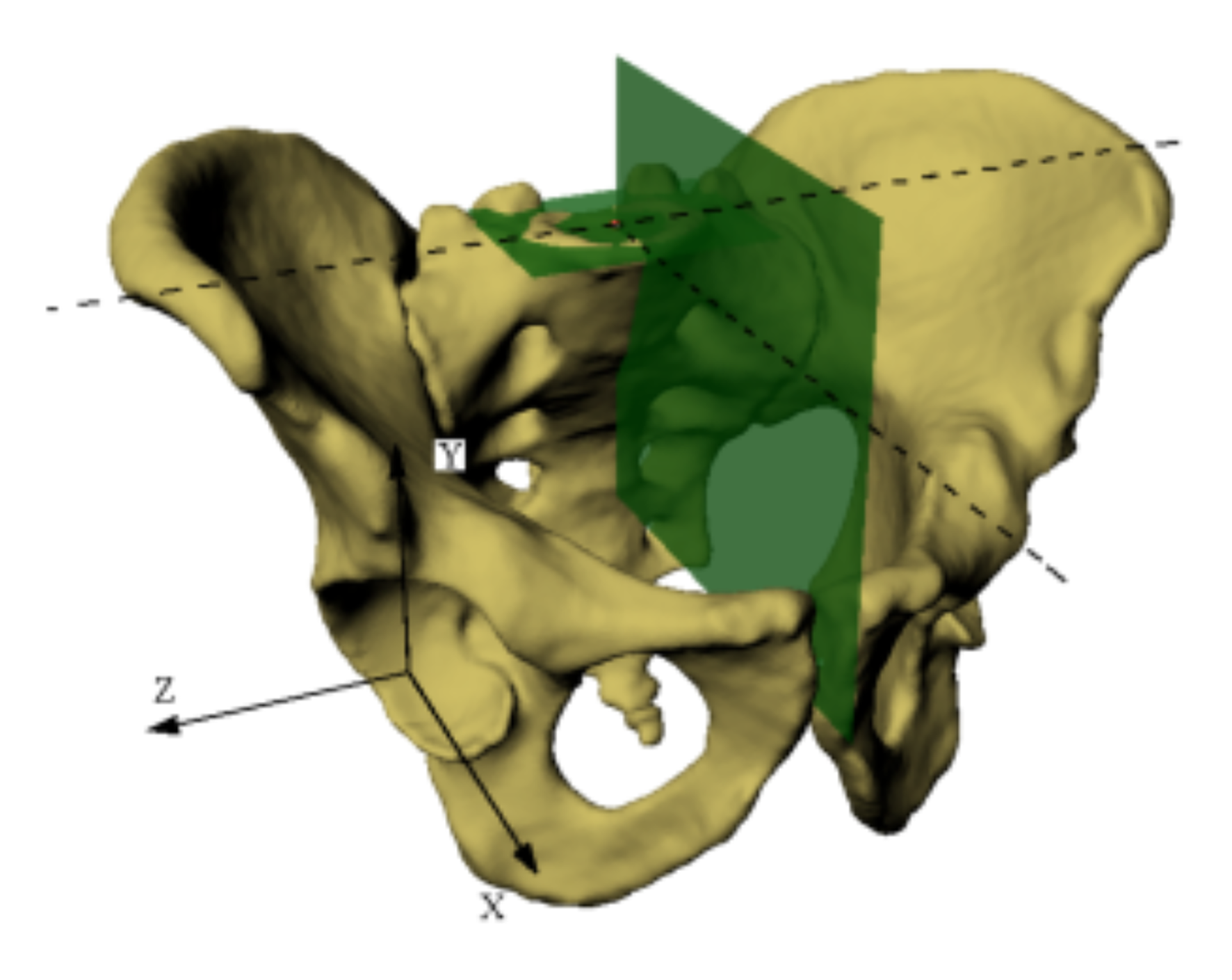

Supplement: Figure S1 [file peerj-07-6433-s001.png]

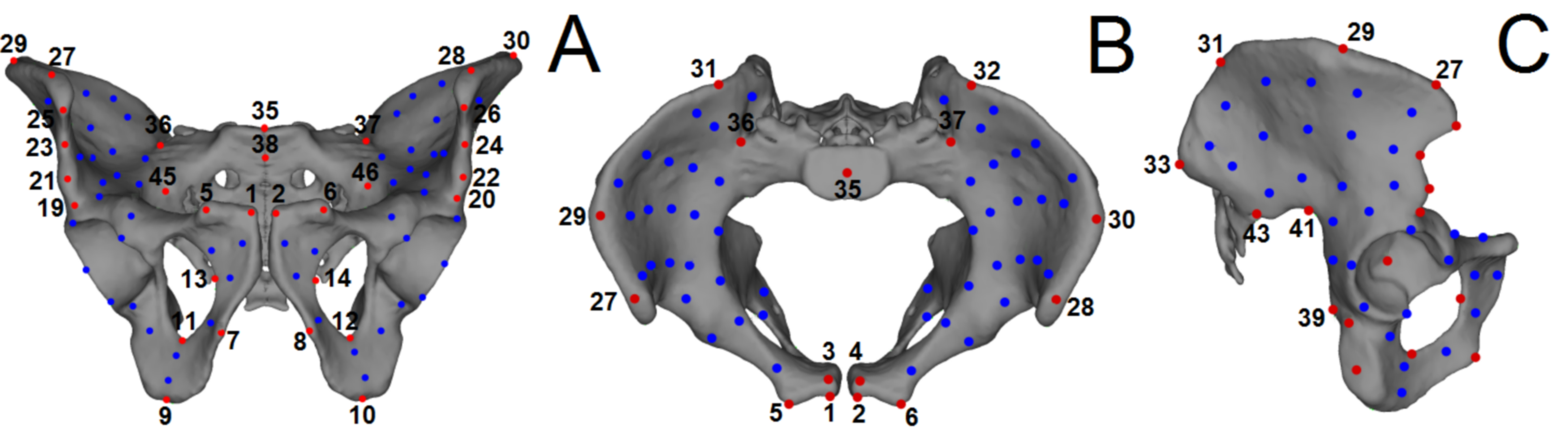

Supplement: Figure S2 — (A) anterior view of the male pelvis; (B) superior view of the male pelvis; (C) lateral view of the male pelvis. [file peerj-07-6433-s002.png]
