# Supplementary material for: Variation in pelvic shape and size in Eastern European males: a computed tomography comparative study
Source: PeerJ. 2019 Feb 20;7:e6433. doi: 10.7717/peerj.6433 (PMC6387581; doi:10.7717/peerj.6433)
Supplement: Table S1 — LM –landmark number [file peerj-07-6433-s003.docx]

Table S1. Definition of landmarks

| LM | Description |
| --- | --- |
| 1-2 | Pubic symphysis: superior-most anterior point |
| 3-4 | Pubic symphysis: superior-most posterior point |
| 5-6 | Tuberculum pubicum |
| 7-8 | Ramus ossis ischii: anterior-most point |
| 9-10 | Tuber ischiadicum: inferior-most point |
| 11-12 | Foramen obturatum: inferior-most point |
| 13-14 | Foramen obturatum: anterior-most point |
| 15-16 | Corpus ossis ischia: most medio-inferior point |
| 17-18 | Acetabulum: deepest point on acetabular fossa |
| 19-20 | Posterior-most point between Spina iliaca anterior inferior and Eminentia iliopubica |
| 21-22 | Spina iliaca anterior inferior: anterior-most point |
| 23-24 | Inferior-most point between spina iliaca anterior superior and spina iliaca anterior inferior |
| 25-26 | Spina iliaca anterior superior: anterior-most point |
| 27-28 | Superior-most point between landmarks 25-26 and 29-30 |
| 29-30 | Crista iliaca: superior-most external point |
| 31-32 | Crista iliaca: superior-most internal point |
| 33-34 | Spina iliaca posterior superior: posterior-most point |
| 35 | S1: superior-most point on S1 center |
| 36-37 | Articulatio sacroiliaca: posterior-most point |
| 38 | S1: anterior midpoint |
| 39-40 | Spina ischiadica: posterior-most point |
| 41-42 | Incisura ischiadica major |
| 43-44 | Spina iliacia posterior inferior |
| 45-46 | Articulatio sacroiliaca: anterior-most point |

LM – landmark number
